# Association between Psychological Status and Functional Outcome in Surgically Managed Fractures around Hip in Geriatric Patients - A Prospective Study

**DOI:** 10.5704/MOJ.2107.004

**Published:** 2021-07

**Authors:** S Bishnoi, N Huda, SMU Islam, A Pant, S Agarwal, R Dholariya

**Affiliations:** Department of Orthopaedics, Teerthanker Mahaveer University Medical College and Research Centre, Moradabad, India

**Keywords:** geriatric, hip fractures, psychological status, depression, anxiety

## Abstract

**Introduction:**

Fractures around the hip in the geriatric population not only lead to functional but also psychological impairment. Psychiatric disturbances can be associated with poor participation in rehabilitation, increased risk of falling again, and higher rates of mortality. The present study was undertaken to assess the association between the psychological status and functional outcome of surgically managed elderly Indian patients who had sustained fractures around the hip.

**Material and Methods:**

The present study was a hospital based prospective, single centre study. One hundred and two geriatric patients who had sustained hip fracture and had been managed surgically, having no cognitive dysfunction, living independently, having unhindered walking capability before the fracture, were included in the study. They were called for follow-up at 3rd, 6th, and 12th month after the hip surgery. Psychological assessment was done by the Hospital Anxiety and Depression Scale (HADS) and functional outcome by using the Harris hip score (HHS).

**Results:**

Our study did not show association between psychological status and functional outcome except in one sub-group. Significant correlation was observed between the psychological status and functional outcome in most of the patients in the extra-capsular group. We have identified improvement in the depression, anxiety and functional scores during the follow-up.

**Conclusion:**

We conclude that psychiatric disturbances in a geriatric patient after undergoing a surgery for hip fracture may lead to poor recovery. We recommend that all such geriatric patients should undergo a psychological assessment and proper therapy should be instituted to achieve good functional recovery.

## Introduction

Geriatric population is not only prone to fractures but also to their adverse outcomes^1-3^. The major factors attributed to the occurrence of these fractures are old age, osteoporosis and frequent falls. These are due to problems in coordination, arthritis, impaired activity of daily living, visual impairment, malnutrition, disability prior to fractures and neuropsychiatric disturbances^4,5^.

Fractures around the hip have been recognised as the common fractures in the elderly and are associated with significant morbidity, mortality and disability^6^. The risk of sustaining a fracture around the hip in a person's lifetime is high, and it ranges from 40% to 50% among females and 13% to 22% among the males^4,6^. In contrast to other general hospital populations, the elderly hip fracture population has high reported rates of psychiatric illness, which is a cause of adverse outcomes. The commonest psychiatric disturbances to be reported in the elderly with fracture around the hip are depression, anxiety and cognitive impairment like delirium and dementia^7,8^. Depression is considered as one of the conditions possibly associated to osteoporotic fractures and studies have reported its association to low bone mineral density^9^.

These psychiatric disorders lead to partial recovery in the psychosocial and physical factors and are found to be more prevalent in patients who sustain fracture around the hip when compared to patients who undergo a surgery which is non-orthopaedic^10^. Depression and anxiety can be associated with poor participation in rehabilitation^8,11^ and increased risk of falling again^7^. It may also affect the recovery of independent walking, lead to higher rates of mortality and increased susceptibility to infectious disease^12^. The prevalence of depression in the elderly following a hip fracture has been reported as varying between 9% and 47%^7^. Despite all the developments in current surgical practices, anaesthesia techniques, post-operative care and rehabilitation, studies revealed that women sustaining a hip fracture had a 5-fold increase and men almost an 8-fold increase in relative likelihood of death within the first three months^13^. In other words, a poor psychological status is associated with adverse outcomes in geriatric patients who undergo surgery for fractures around the hip. Though there are many studies which have established this association, most of these have been reported in the Western literature.

The main aim of the present study was to (1) find out the association between psychological status (depression, anxiety) and functional outcome, (2) find out the correlation between psychological status (depression, anxiety) and functional outcome and (3) identify the changes in the psychological status (depression, anxiety) and functional outcome in surgically managed fractures around the hip in Indian geriatric patients.

## Materials and Methods

The present study was a hospital based prospective, single centre study conducted between January 2017 and September 2019. One hundred and two geriatric patients who had sustained a fracture around the hip joint and had been managed surgically, of age >60 years, having no cognitive dysfunction (assessment was done by using the Mini-Mental State Examination and 4 'A's Test), were living independently, having unhindered walking capability before the fracture, were included in the study. Patients having multiple fractures, any pathological fracture other than fragility fracture, those who were visually handicapped or had cognitive dysfunction were excluded from the study.

An informed written consent was taken from all the patients willing to participate in the study on an Informed consent form (ICF) as per the guidelines of Institutional Ethical Committee (IEC). The protocol, case report form and informed consent were approved by the Institutional Ethical Committee.

**Sample Size:** Sample size based on estimation of Mean,

$$n={\left(\frac{{}^{z}\alpha /2^{\sigma }}{d}\right)}^{2}$$

Where the following applies:

σ= Standard deviation

d= Precision

= Confidence level alpha (α) =1.96

σ= 9.3

d=2

n=83 (Calculated sample size)

Total obtained sample size was 92.

All the patients included in the study were divided into two groups, Group A and Group B. Patients of Group A were cases of intracapsular fractures and patients of Group B included cases of extracapsular fractures. Group A was further subdivided into two sub-groups, A1 and A2. All cases of fresh un-displaced fracture neck of femur fell into group A1 and all cases of old (>3 weeks) / displaced fracture neck of femur fell into group A2. Similarly, patients of the stable intertrochanteric fracture fell into group B1 and cases of the unstable intertrochanteric fracture fell into group B2. The patients in group A1 were treated using osteosynthesis techniques like three Cannulated Cancellous screw / Dynamic Hip Screw (DHS) and patients in group A2 were managed by performing Hemi-arthroplasty with Austin Moore Prosthesis (AMP) / Bipolar prosthesis. Patients in B1 group were treated using Dynamic hip screw / Proximal femoral nail (PFN) and patients in group B2 were treated using PFN / PFNA.

Quadriceps exercises and calf pumps were initiated as early as the patient was weaned off from the effects of anesthesia. Full range of motion exercises for knee and ankle was started from day one. In sub-group A1, B1 and B2 toe Touchdown weight bearing was started from day three by using a walking frame and continued up to six weeks, followed by full weight bearing depending on the fracture union. In subgroup A2 patient’s full weight bearing was initiated at the 3rd post-operation day depending on the pain tolerance of the patient. The functional outcome was assessed using the Harris hip score^14^ and the psychological outcome was assessed using the HADS^15^ score at 3rd, 6th, and 12th month after the surgery. The HHS is an outcome scoring system dependent on the clinicians. The questionnaire has 10 questions and score ranges from 0-100 with higher values exhibiting good functions and better results. A score between 90-100 is graded excellent, 80-89 is a good score, 70-79 is fair score and a score less than 70 is a poor score. The hospital anxiety and depression scale (HADS)^15^ assess the anxiety and depression on a scale of 0-21 score separately. A score between 0-7 is considered normal, 8-10 is borderline abnormal and 11-21 is abnormal.

SPSS 20.0 Version was used for statistical analysis. One-way ANOVA was computed to find the significant association between psychological status (depression, anxiety) and functional outcome. Pearson’s correlation co-efficient was used to find the correlation between psychological status (depression, anxiety) and functional outcome among participants in different groups. Repeated measures of ANOVA were computed to observe changes, in the score of psychological status (depression, anxiety scores) and the functional outcome.

## Results

A total of 102 patients were included in the study after they had fulfilled the inclusion criteria. Patients who were either lost to follow-up or in whom complications like non-union was observed and required secondary operative procedure were not included in the final analysis. The final data analysis was performed on 92 patients only. The mean age of participants was 74.59±10.11 years. Out of the 92 patients, 56 were males and 36 were females. Demographic characteristics and distribution of the Patients in different sub-groups are shown in Table I. For the final analysis there were 23 patients in sub-group A1, 34 patients in sub-group A2, 14 patients in B1 and 21 patients in B2 sub-group.

**Table I:** Demographic characteristics and distribution of the patients

	Sub-group A1	Sub-group A2	Sub-group B1	Sub-group B2
Number of Patients	n = 23	n = 34	n = 14	N = 21
Male	14	20	9	13
Female	9	14	5	8
Mean Age (years)	72.37 ± 8.30	76.54 ± 10.12	74.24 ± 11.2	78.2 ± 9.2
Side- Right/ Left	12/11	21/13	6/8	11/10
Body Mass Index (BMI)	27.2 ± 3.6	30.7 ± 4.4	28.2 ± 4.2	29.32 ± 3.2
Duration of hospital stay (Days)	8.2 ± 3.2	7.32 ± 2.59	9.04 ± 2.99	8.42 ± 1.8

One-way ANOVA was computed to find the significant association between psychological status (depression, anxiety) and functional outcome. It revealed that there was no statistically significant association observed between psychological status (depression, anxiety) and functional outcome among participants in different sub-groups at p>0.05, except the B2 sub-group for depression and their functional outcome at p=0.01 (Table II).

**Table II:** Association between psychological status (depression, anxiety) and functional outcome among Participants in different sub-groups

	Psychological Status		n	Functional Outcome M	SD	F value, df, p value
1.	A1 sub-group (Depression)	Border Abnormal (8-10)	2	61.50	19.09	F= 0.90
		Abnormal (11-21)	21	69.19	10.37	df=1
						p= 0.35(NS)
	A1 sub-group (Anxiety)	Normal (0-7)	22	68.23	11.08	F= 0.35
						df=1
		Border Abnormal (8-10)	1	75.00	----	p= 0.55(NS)
2.	A2 sub-group (Depression)	Normal (0-7)	24	81.13	10.62	F= 2.14
		Border Abnormal (8-10)	9	87.11	8.08	df=2
						p= 0.13(NS)
	A2 sub-group (Anxiety)	Abnormal (11-21)	1	97.00	----	
		Border Abnormal (8-10)	33	82.76	10.24	F= 1.87
		Abnormal (11-21)	1	97.00	----	df=1
						p= 0.18(NS)
3.	B1 sub-group (Depression)	Normal (0-7)	8	87.63	6.39	F= 0.14
		Border Abnormal (8-10)	6	88.00	5.29	df=1
						p= 0.90(NS)
	B1 sub-group (Anxiety)	Normal (0-7)	13	87.77	5.96	F= 0.01
		Border Abnormal (8-10)	1	88.00	----	df=1
						p= 0.97(NS)
4.	B2 sub-group (Depression)	Normal (0-7)	17	68.76	9.78	F= 7.78
		Border Abnormal (8-10)	4	82.75	2.21	df=1
						p= 0.01(S)
	B2 sub-group (Anxiety)	Normal (0-7)	7	66.57	12.6	F= 1.72
		Border Abnormal (8-10)	5	70.20	11.45	df=2
						p= 0.20 (NS)
		Abnormal (11-21)	9	75.89	6.56	

p<0.05- significance level, S:Significant, NS: Non-Significant

The correlation between psychological status (depression, anxiety) and functional outcome among participants in different sub-groups (Table III) was computed with the help of Pearsons correlation co-efficient and it reveals that there was a weak positive correlationship between depression scores and Harris Hip scores in A1 sub-group, ‘r’ value is 0.13 and it was not statistically significant (p=0.54) and there was a weak positive correlationship between anxiety scores and Harris Hip scores in A1 sub-group, ‘r’ value is 0.26 and it was not statistically significant (p=0.21). There was a moderate positive correlationship between depression Scores and Harris Hip scores in A2 sub-group, ‘r’ value was 0.49 and it was statistically significant (p=0.003) and there was a moderate positive correlationship between anxiety scores and Harris Hip scores in A2 sub-group, ‘r’ value was 0.16 and it was not statistically significant (p=0.35).

**Table III:** Correlation between psychological status (depression, anxiety) and functional outcome among participants in different sub-groups

Variable		Respondents		Correlation and coefficient (r) value	p value
		Mean	Standard deviation		
A1 sub-group	Depression Scores	12.74	1.73	0.13	0.54(NS)
	Harris Hip Scores	68.52	10.92		
	Anxiety Scores	4.22	1.80	0.26	0.21(NS)
	Harris Hip Scores	68.52	10.92		
A2 sub-group	Depression Scores	6.12	2.47	0.49	0.003(S)
	Harris Hip Scores	83.18	10.37		
	Anxiety Scores	2.97	2.03	0.16	0.35(NS)
	Harris Hip Scores	83.18	10.37		
B1 sub-group	Depression Scores	6.86	2.14	-0.18	0.52(NS)
	Harris Hip Scores	87.79	5.72		
	Anxiety Scores	4.29	1.63	0.81	0.0001(S)
	Harris Hip Scores	87.79	5.72		
B2 sub-group	Depression Scores	9.52	3.82	0.43	0.04(S)
	Harris Hip Scores	71.43	10.43		
	Anxiety Scores	5.81	1.96	0.53	0.01(S)
	Harris Hip Scores	71.43	10.43		

*significant relationship (p< 0.05)

There was a weak negative correlationship between depression scores and Harris Hip scores in B1 sub-group, ‘r’ value is -0.18 and it was not statistically significant (p=0.52) and there was a strong positive correlationship between anxiety scores and Harris Hip scores in B1 sub-group, ‘r’ value is 0.81 and it was statistically significant (p=0.001). There was a moderate positive correlationship between depression scores and Harris Hip scores in B2 sub-group, ‘r’ value is 0.43 and it was statistically significant (p=0.04) and there was a moderate positive correlationship between anxiety scores and Harris Hip scores in B2 sub-group, ‘r’ value was 0.53 and it was statistically significant (p=0.01).

The data presented in the Table IV shows the repeated measures of ANOVA computed for psychological status (depression, anxiety scores) and functional outcome (Harris Hip Scores) between the sub-group A1 and A2. For depression scores (F=11.11, p=0.002) there was gradual decrease in depression scores in A2 sub-group compared to A1 sub-group and the changes observed from pre-operative to post-operative periods were statistically significant. For anxiety scores (F=14.19, p=0.001) there was gradual decrease in anxiety scores in A2 sub-group compared to A1 sub-group and the changes observed from pre-operative to post-operative periods were statistically significant. For Harris Hip scores (F=27.11, p=0.001) there was gradual improvement in functional scores (Harris Hip Scores) in A2 sub-group compared to A1 sub-group and the changes observed in different post-operative periods were statistically significant. In A2 sub-group HHS had improved from poor to good at final follow-up while in A1 sub-group it remained poor at final follow-up. Depression and anxiety scores were abnormal or borderline abnormal in sub-group A2 initially, which became normal at 12th month follow-up. Depression score in sub-group A1 had changed from borderline abnormal to abnormal at final follow-up and anxiety scores had shifted towards normalcy at 12th month follow-up.

**Table IV:** Repeated measures of ANOVA on psychological status (depression, anxiety scores) and functional outcome (Harris Hip Scores) among participants in A1 sub-group and A2 sub-group at all-time points

Variable		A1 sub-group n=23		A2 sub-group n=34		Between the sub-group *F* Value*(df) p* value
		M	SD	M	SD	
Depression Scores	Pre-operative	7.74	1.57	8.18	3.63	*F*=11.11
	Post-operative at 3rd Month	9.43	1.53	9.47	3.37	*df*=1
	Post-operative at 6th Month	10.09	1.50	7.94	2.71	*p*=0.002(S)
	Post-operative at 12th Month	12.74	1.73	6.12	2.47	
Anxiety Scores	Pre-operative	13.39	2.33	9.88	3.64	*F*=14.19
	Post-operative at 3rd Month	9.30	2.28	8.35	2.67	*df*=1
	Post-operative at 6th Month	7.65	2.22	5.79	2.82	*p*=0.0001(S)
	Post-operative at 12th Month	4.22	1.80	2.97	1.37	
Harris Hip Scores	Post-operative at 3rd Month	53.26	10.81	69.53	14.23	*F*=27.11
	Post-operative at 6th Month	62.70	9.29	77.65	12.05	*df*=1
	Post-operative at 12th Month	68.52	10.92	83.18	10.37	*p*=0.0001(S)

(p<0.05 significant level) S-Significant

The data presented in Table V shows that the repeated measures of ANOVA computed for psychological status (depression, anxiety scores) and functional outcome (Harris Hip Scores) between the sub-group B1 and B2. For depression scores (F=1.95, p=0.17) there was not much difference identified in depression scores between B1 subgroup compared to B2 sub-group and the changes observed from pre-operative to post-operative periods were not statistically significant. For anxiety scores (F=1.26, p=0.26) there was not much difference identified in anxiety scores in B1 sub-group compared to B2 sub-group and the changes observed from pre-operative to post-operative periods were not statistically significant. For Harris Hip scores (F=13.26, p=0.001) there was gradual improvement in functional scores (Harris Hip Scores) in B1 sub-group compared to B2 sub-group and the changes observed in different post-operative periods were statistically significant. In B1 subgroup HHS had improved from fair to good at final follow-up while in B2 sub-group poor to fair at final follow-up.

**Table V:** Repeated measures of ANOVA on psychological status (depression, anxiety scores) and functional outcome (Harris Hip Scores) among participants in B1 sub-group and B2 sub-group at all-time points

Variable		B1 sub-group n=14		B2 sub-group n=21		Between the sub-group *F* Value*(df) p* value
		M	SD	M	SD	
Depression Scores	Pre-operative	8.29	2.43	8.62	3.20	*F*=1.95
	Post-operative at 3rd Month	11.43	2.53	11.43	3.12	*df*=1
	Post-operative at 6th Month	8.50	2.65	10.86	3.36	*p*=0.17(NS)
	Post-operative at 12th Month	6.86	2.14	9.52	3.82	
Anxiety Scores	Pre-operative	11.07	2.75	11.71	2.43	*F*=1.26
	Post-operative at 3rd Month	8.64	2.67	9.10	2.32	*df*=1
	Post-operative at 6th Month	6.71	2.70	7.33	2.26	*p*=0.26(NS)
	Post-operative at 12th Month	4.29	1.63	5.81	1.96	
Harris Hip Scores	Post-operative at 3rd Month	74.50	12.13	64.71	13.33	*F*=13.26
	Post-operative at 6th Month	81.79	9.79	67.95	11.54	*df*=1
	Post-operative at 12th Month	87.79	5.72	71.43	10.43	*p*=0.001(S)

(p<0.05 significant level) S-Significant, NS: Non-significant

## Discussion

Neuropsychiatric complications have always been a great concern in elderly patients after hip fracture surgeries. These conditions are not only related to their poor functional recovery but also to the increased mortality. Our study on surgically managed fractures around the hip in Indian geriatric patients has not shown significant association between psychological status and functional outcome, except the B2 sub-group of patients with unstable intertrochanteric fracture, where significant correlation was observed between the psychological status and functional outcome in most of the patients in the extra-capsular group. Our study has identified an improvement in the depression, anxiety and functional scores during the follow-up period except in A1 sub-group of patients with fresh undisplaced fracture neck of femur, which have shown an increasing trend in depression scores. We have observed significant improvement in the psychological and functional scores of the patients with fresh displaced fracture neck of femur (sub-group A2) as compared to the patients with fresh non-displaced fracture neck of femur (sub-group A1) during follow-up. The improvement was not significant in terms of psychological scores, but functional scores were observed to be significant in patients with the unstable intertrochanteric fracture (subgroup B2) as compared to patients with the stable intertrochanteric fracture (sub-group B1) during follow-up.

Past studies have reported the significance of psychological assessment of the patients undergoing surgeries around the hip and their role in the functional outcome. Benditz *et al* (2017)^16^ conducted a study with 50 patients after primary unilateral THA and observed a significant relationship of the changes in anxiety and depression scores with hip functionality. The scale used was the general depression scale known as ADS-L; in the pre-operative period the score was 16.8±8.8 which decreased to 11.9±6.2, five weeks after the operation and the state anxiety was 44.1±12.3 at the start and thereafter, decreased to 35.1±10.2. The Harris Hip score was increased from 49.6±19.8 to 73.3±8.8. They emphasise the important role of psychological factors on the outcome^16^ (Table VI). In our study we have also observed correlation between the psychological status and functional outcome in the study population. In all sub-groups the Harris hip score was improved to fair or good at final follow-up; depression and anxiety scores were in the range of abnormal or borderline abnormal initially, which has shifted towards normalcy at 12th month follow-up in most of the patients.

**Table VI:** Characteristics of patients and its relationship with psychological and/or functional status

Study	Total patients	Age/Sex	Fracture pattern/Mx	Depression				Anxiety				Functional status		
				HADS-D (D) (Mean Value)				HADS-A (A) (Mean Value)				Harris hip score(h) (Mean Value)		
Present study	N = 92	Mean age - 74.59 ± 10.11	A1 = 23	Pre-op	3 months	6 months	12 months	Pre-op	3 months	6 months	12 months	3 months	6 months	12 months
	Group A = 57	years		7.74	9.43	10.09	12.74	13.39	9.30	7.65	4.22	53.26	62.7	68.52
	Group B = 35	M = 56	A2 = 34	8.18	9.47	7.94	6.12	9.88	8.35	5.79	2.97	69.53	77.65	83.18
		F = 36	B1 = 14	8.29	11.43	8.50	6.86	11.07	8.64	6.71	4.29	74.5	81.79	87.79
			B2 = 21	8.62	11.43	10.86	9.52	11.71	9.10	7.33	5.81	64.71	67.95	71.43
					HAM-D (Mean Value)				STAI-Y1 (Mean Value)			Physical Functioning( SF-36)		
Gambatesa et al^17^	N = 40	Mean age - 81.3	Intracapsular 7		T0	T2	Significance		T0	T2	Significance	T0	T2	Significance
	Group C = 20	M = 1	Extracapsular 13		15.7	5.7	P<0.01	54.1		45	P<0.05	53.5	75.3	P<0.05
		F = 19												
	Group NC = 20	Mean age - 80.3	Intracapsular 5		11.2	10.9	NS		49	49	NS	45.8	70.3	P<0.05
		M =2	Extracapsular 15											
		F = 18												
					ADS-L (D) (Mean value)				STAI-X1 (A) (Mean value)			Harris hip score(h) (Mean value)		
Benditz et al^16^	N = 50	Mean age - 62.18 years	Total Hip		T0	T1	T2		T0	T1	T2	T0	T1	T2
		M = 23	Arthroplasty		16.80	17.58	11.90		44.06	38.88	35.14	49.66	60	73.28
		F = 27												
					Depression Anxiety Stress Scale- Depression subscale (Mean value)				Depression Anxiety Stress Scale – Anxiety subscale (Mean value)					
Bruggemann et al^8^	N = 103	Mean age - 78.04	Hip fracture		T1	T2	Significance		T1	T2	Significance	NA		
		M = 22			2.67	2.93	P=0.489		4.58	2.92	P=0.001			
		F=81													

Another study by Gambatesa *et al* (2013)^17^ on forty patients who were managed surgically for the hip fracture were divided into two sub-groups, one received counselling and the control without counselling. Short Form-36-item Health Survey Questionnaire, State–Trait Anxiety Inventory and Hamilton Rating Scale for Depression scores were recorded and the patients undergoing counselling showed decreasing HAMS-D scores and STAI-Y1 scores after 30 days when compared to the baseline evaluation. They remained stable in the non-counselling group, but physical functioning improved in all patients. The scores in non-counseling group correlate with our study. They also stated that, it is an obsolete and wrong conception that treatment ends with the surgical procedure and physical rehabilitation, because a patient’s psychological status plays a critical role in regaining good performance levels^17^ (Table VI). Bruggemann *et al* (2007) investigated the role of injury-related beliefs and hopelessness on depression and anxiety in 103 hip fracture patients at two time points and observed that approximately one month following injury, 16% and 25% of the sample had moderate or higher levels of depressive and anxiety symptoms, respectively. There appeared to be an improvement in anxiety symptoms and depression remained considerably stable relative to the first assessment. The results were comparable to our study. They had supported a possibility for psychological intervention on the basis, of their findings^8^ (Table VI).

With the increase in the elderly population, health status of the elderly gained lots of importance and there is evidence of a rise in morbidity, mortality and loss of functional status which are related to psychological conditions in elderly patients. We recommend that, every geriatric patient of fracture around the hip should undergo a regular neuropsychiatric assessment targeted to their mental health needs. The limitation of the present study is that it was a single centre study and sample size was small, due to which generalisation of the study population may not be possible.

## Conclusion

Studies have proved the existence of depression and anxiety in the post-operative period, and it is more evident to geriatric patients. In our study on surgically managed fractures around the hip in Indian geriatric patients a significant correlation was observed for depression and anxiety with the functional outcome in many of the patients; it was also observed that patient's depression and anxiety scores improve with the functional outcome. Thus, we conclude that these psychiatric disturbances in a geriatric patient after undergoing a surgery for the hip fracture may lead to a poor recovery. We recommend that all such geriatric patients should undergo a psychological assessment to recognise their special needs and proper therapy should be instituted in order, to achieve a good functional recovery.
